# Functional recovery after treatment of extra-articular distal radius fractures in the elderly using the IlluminOss® System (IO-Wrist); a multicenter prospective observational study

**DOI:** 10.1186/s12891-016-1077-9

**Published:** 2016-05-27

**Authors:** Tjebbe Hagenaars, Guido W. Van Oijen, W. Herbert Roerdink, Paul A. Vegt, Jos P. A. M. Vroemen, Michael H. J. Verhofstad, Esther M. M. Van Lieshout

**Affiliations:** Trauma Research Unit Department of Surgery, Erasmus MC, University Medical Center Rotterdam, P.O. Box 2040, Rotterdam, CA 3000 The Netherlands; Department of Surgery, Deventer Hospital, P.O. Box 5001, Deventer, GC 7400 The Netherlands; Department of Surgery, Albert Schweitzer Hospital, P.O. Box 444, Dordrecht, AK 3300 The Netherlands; Department of Surgery, Amphia Hospital, P.O. Box 90158, Breda, RK 4800 The Netherlands

**Keywords:** Costs, Distal radius fracture, Elderly, Extra-articular, Fracture, Intramedullary, Outcome

## Abstract

**Background:**

Approximately 17 % of all fractures involve the distal radius. Two-thirds require reduction due to displacement. High redislocation rates and functional disability remain a significant problem after non-operative treatment, with up to 30 % of patients suffering long-term functional restrictions. Whether operative correction is superior to non-operative treatment with respect to functional outcome has not unequivocally been confirmed. The IlluminOss® System was introduced in 2009 as a novel, patient-specific, and minimally invasive intramedullary fracture fixation. This minimally invasive technique has a much lower risk of iatrogenic soft tissue complications. Because IlluminOss® allows for early mobilization, it may theoretically lead to earlier functional recovery and ADL independence than non-operative immobilization. The main aim of this study is to examine outcome in elderly patients who sustained a unilateral, displaced, extra-articular distal radius fracture that was treated with IlluminOss®.

**Methods/design:**

The design of the study will be a multicenter, prospective, observational study (case series). The study population comprises elderly (60 years or older; independent in activities of daily living) with a unilateral, displaced, extra-articular distal radius fracture (AO/OTA type 23-A2 and A3) that after successful closed reduction was fixed within 2 weeks after the injury with IlluminOss®. Critical elements of treatment will be registered, and outcome will be monitored until 1 year after surgery. The Disabilities of the Arm, Shoulder, and Hand score will serve as primary outcome measure. The Patient-Rated Wrist Evaluation score, level of pain, health-related quality of life (Short Form-36 and EuroQoL-5D), time to ADL independence, time to activities/work resumption, range of motion of the wrist, radiological outcome, and complications are secondary outcome measures. Health care consumption and lost productivity will be used for a cost analysis. The cost analysis will be performed from a societal perspective. Descriptive data will be reported.

**Discussion:**

The results of this study will provide evidence on the effectiveness of operative treatment of patients who sustained an extra-articular distal radius fracture with the IlluminOss® System, using clinical, patient-reported, and societal outcomes.

**Trial registration:**

The study is registered at the Netherlands Trial Register (NTR5457; 29-sep-2015).

## Background

Distal radius fractures are the second most common osteoporotic fractures [1]. The number of hospitalizations due to these fractures in The Netherlands in patients aged 50 years and older increased from 877 in 1997 to 2912 in 2009 [2]. In line with demographic developments, osteoporotic fractures including those of the wrist can be expected to increase further in the coming years, with a concomitant increased burden on health care resources [3–5].

The current standard treatment for patients with a displaced extra-articular distal radius fracture as mentioned in the Dutch treatment guideline is closed reduction and plaster cast immobilization for 4–6 weeks [6]. Non-operative treatment remains controversial due to high dislocation rates (36–57 %) and often disappointing functional recovery [6–11]. Immobilization may also induce stiffness. Multiple studies have shown that comminuted extra-articular distal radius fractures are characterized by instability, and plaster immobilization will usually not prevent substantial redisplacement [8, 12, 13].

Several operative procedures are used for extra-articular distal radius fractures. In osteoporotic bone, angular stable plates are widely used, whereas the interest in and use of intramedullary devices is growing. Potential advantages of modern intramedullary devices could be the minimal surgical exposure for introduction of the device and the low risk of implant-related soft tissue irritation. This includes a reduced risk of tendon injury and screw penetration into the joint, which are the main problems of volar plating [14]. A recent literature review showed that intramedullary nailing gives a comparable range of motion, functional outcome, and grip strength as other fixation techniques, however the 0–50 % complication rate may raise concerns about the role of intramedullary nailing [15]. In addition to injury of the superficial radial nerve as most common complication (occurring in 9.5 % of patients), irritation from metalwork, tenosynovitis, and infection occurred in 1-2 % of patients. New devices and surgical techniques should be aimed at reducing the risk of these complications.

The llluminOss® System is the first percutaneous, patient-specific, fracture stabilization system [16]. Only a small and percutaneous incision is required to insert an inflatable balloon into the medullary canal. The balloon, which spans the fracture is infused with a light curable monomer. The monomer hardens in situ by applying a visible light, resulting in a stable and patient-specific implant that provides longitudinal strength and rotational stability over the length of the implant. Early functional after-treatment without additional plaster immobilization is possible. The latter is considered the main strength of this device over non-operative treatment and conventional metal implants [16].

The IO-WRIST study is designed to determine results after treatment with IlluminOss®. Outcome will be monitored as propagated by the distal radius working groups of the International Society for Fracture Repair and the International Osteoporosis Foundation [17]. This study primarily aims to examine the effect of operative treatment using the IlluminOss® System on the DASH (Disabilities of the Arm, Shoulder, and Hand) score in elderly patients who sustained a unilateral, displaced, extra-articular distal radius fracture. Secondary objectives are to examine the effect on functional outcome, the level of pain, health-related quality of life, the time to regaining independence in activities of daily living (ADL), the resumption of work/ADL, range of motion of the wrist, the time to radiographic healing, the rate of complications with associated secondary interventions, and the costs for health care use and lost productivity.

## Methods/design

### Study design

The IO-Wrist study is a multicenter, prospective, observational study (i.e., case series). As the intervention studied is a relatively new technique and there is a lack of published evidence a case series was preferred over a study comparing the intervention with a control group. The following four hospitals in The Netherlands will participate: 1) Albert Schweitzer Hospital, Dordrecht; 2) Amphia Hospital, Breda; 3) Deventer Hospital, Deventer; and 4) Erasmus MC, Rotterdam. The study is registered at the Netherlands Trial Register (NTR5457), registration date September 29, 2015.

### Recruitment and consent

Eligible persons who present to the Emergency Department (ED) with a unilateral, displaced, extra-articular distal radius fracture will be informed about the study after the fracture has been reduced or as early as possible after operative treatment. They may receive the information at the ED or upon admission at the surgical ward. The clinical investigator, an assistant, or the attending physician will explain the study, and give the patient written information and a consent form. Patients meeting all eligibility criteria will be included as soon as possible. This can be done while patients are still at the ED or when they return to the outpatient department at 2 weeks after surgery.

In order to reduce bias as much as possible, the follow-up measurements by the clinical investigator or research assistant will be performed using a standardized protocol. Evaluation of fracture healing from radiographs will be done by two (independent) trauma surgeons independently. Any disagreement will be discussed and until consensus is reached.

### Study population

All elderly persons aged 60 years or older, who were independent in activities of daily living before the injury, presenting to the ED with a unilateral, displaced, extra-articular distal radius fracture (AO/OTA type 23-A2 and A3) [18] and treated with IlluminOss® will be considered eligible for inclusion. The fracture will be classified on lateral (preferably lateral carporadial) and anteroposterior (AP) radiographs.

The inclusion criteria are:

1. Adult men or women with an age of 60 years or older
2. A unilateral, displaced, extra-articular distal radius fracture (AO/OTA type 23-A2 or A3), as confirmed on X-ray (lateral (preferably lateral carporadial) and AP)
3. Capable of independent activities of daily living prior to index injury*
4. Closed reduction and intramedullary fixation using the IlluminOss® System within 14 days after trauma
5. Provision of informed consent by patient.

* This will be assessed using a simple yes/no question concerning their independence.

The exclusion criteria are:

1. Additional traumatic injuries if this affects treatment, rehabilitation, or function of the affected wrist
2. A pathological, recurrent, or open (i.e., Gustilo-Anderson grade II or III) fracture
3. An impaired wrist function at the affected side) due to arthrosis, rheumatoid disorder, or neurological disorder prior to the injury
4. A bone disorder (excluding osteoporosis), that may impair bone healing (e.g., osteomalacia, renal osteodystrophy, or Paget’s disease)
5. Patient is unwilling or unable to comply with the after-care protocol and follow-up visit schedule
6. Insufficient understanding of the Dutch language for adequate comprehension of the treatment information or rehabilitation program, as judged by the attending physician or researcher
7. Participation in another surgical intervention or drug study.

### Intervention

**Fig. 1:**
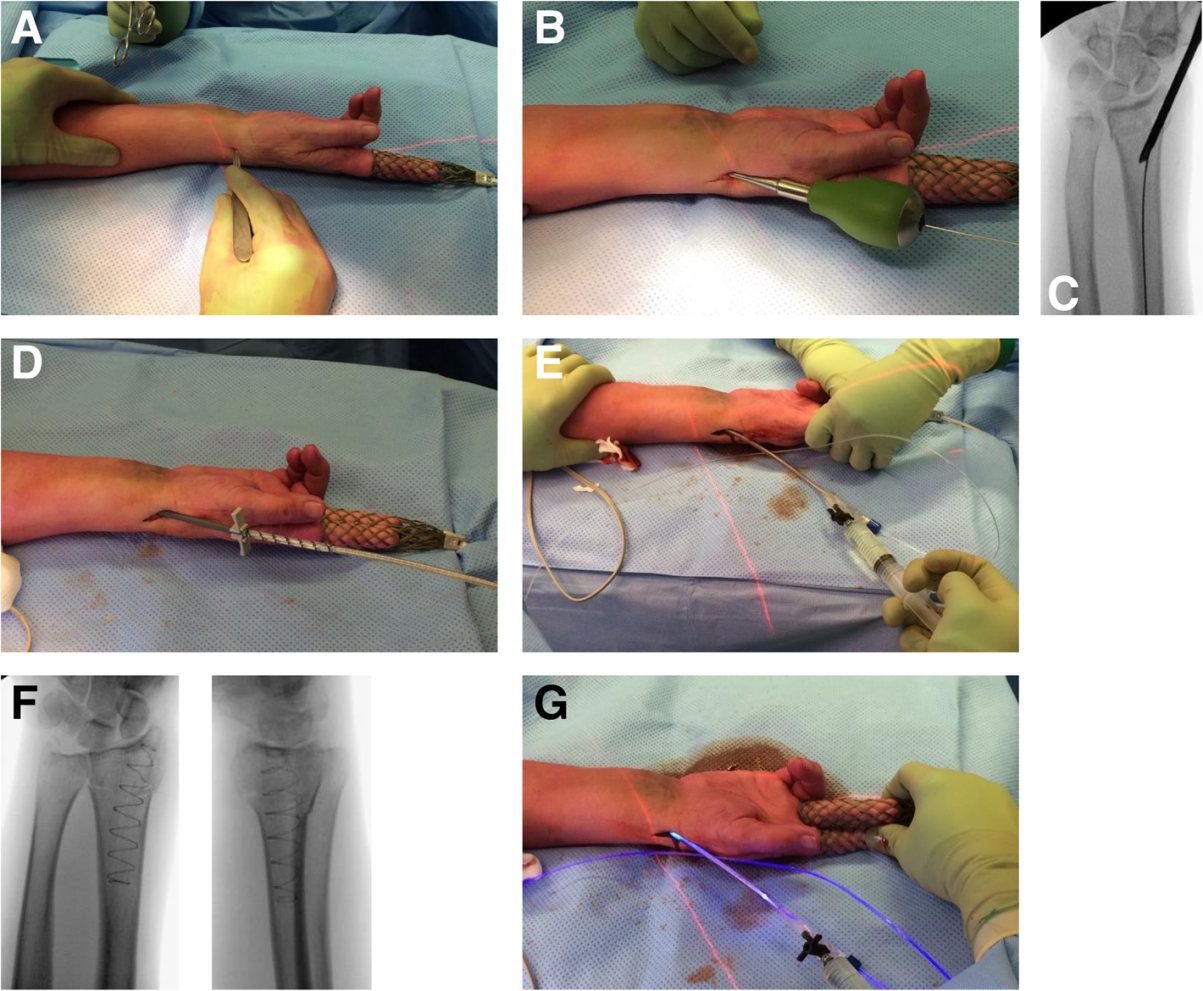
Using the IlluminOss® System for fixating a distal radius fracture*. **a** 1.5–2.0 cm incision over the radial styloid process, between the first and second extensor compartment to reach the periosteum. The superficial branches of the radial nerve are protected. **b** Access to the medullary canal and insertion of a 1.5 mm guide-wire. **c** Correct position is verified by intra-operative fluoroscopy. **d** Flexible balloon catheter is placed intramedullary over the guide-wire spanning the fracture. **e** Infusion of liquid monomeric material and expansion of the balloon conforming to the patient’s unique medullary canal. **f** Verification of adequate fracture reduction, correct balloon position, and balloon expansion. **g** Polymerization (hardening) of the infused monomer by applying visible (436 nm) light, creating a patient specific intramedullary implant. * (Photographs and radiographs are used with permission of the patient)

Following successful closed reduction, patients will undergo fixation with the IlluminOss® System (IlluminOss® Medical, East Providence, RI, USA), using a standardized protocol and the manufacturer’s guidelines (Fig. 1). During the procedure, the fracture is reduced and stabilized. A customized, inflatable, thin walled polyethylene terephthalate (PET; Dacron) balloon is mounted on an insertion catheter. The small diameter compressed balloon (~3.5 mm) is inserted into the medullary canal spanning the fracture site. Next, the delivery sheath is removed, uncovering the balloon. The balloon is then infused with a biocompatible photodynamic liquid monomer via a syringe, causing the balloon to expand. The inflated implant conforms to the shape of the canal and curves from the entry at the radial styloid into the canal. The formed arc within the radial box acts as a support structure to the floor of the articular surface and contributes to a transverse reduction force. The bone is visualized under fluoroscopy to ensure that the fracture has been properly reduced, with the balloon in the appropriate position, fully inflated and contacting the inner diameter of the bone. Upon activation of the light system (Blue Wave 75 VT Photodynamic Light Box), the photo initiator in the monomer rapidly polymerizes and hardens in situ to form a strong hardened nail that provides articular support and canal and rotational stability. This aids fracture healing by primary callous formation and remodeling.

Patients will receive antibiotics prophylaxis according to the local protocol. They are discharged with either a double bandage or a soft cast. No post-operative mobility restrictions will apply, and patients will be allowed to use their wrist as tolerated immediately.

Relevant details of surgery will be collected (e.g., length and diameter of the balloon, time between injury and surgery, duration of the operation) for assessing the effect on outcome. There will be no restriction in the use of (analgetic) medication or other kind of intervention. Details on the use of analgesics will be recorded. Details of physical therapy and rehabilitation will also be recorded. They will not be standardized as evidence favoring a particular approach is not available. Bone growth stimulators (electrical, ultrasound, or magnetic) are prohibited.

All participating surgeons have attended a technique-oriented training course and performed multiple procedures before commencing the study.

### Outcome measures

The primary outcome measure is the Disabilities of the Arm, Shoulder and Hand outcome measure (DASH). This questionnaire is validated and is completed by patients. It reflects the disability experienced by patients with an upper-limb disorder, and is used for monitoring changes in symptoms and function over time [19–22]. The DASH disability/symptom score is the sum of responses to 30 items (scored 1-5), which test a) the degree of difficulty in performing a variety of physical activities (21 items); b) the severity of pain, activity-related pain, tingling, weakness, and stiffness (5 items); and c) the effect on social activities, work, sleep, and self-image (4 items). The total score ranges from zero (no disability) to 100 points (severe disability). At least 27 of the 30 items must be completed. A validated Dutch version is available [23]. The DASH optional modules for Work and high performance Sport/Music (both 4 items, scored 1–5) will also be completed. The score can only be calculated if all items are completed.

The secondary outcome measures are:

- Patient-Rated Wrist Evaluation (PRWE)
- Pain level at both sides (VAS)
- Health-related quality of life: Short Form-36 (SF-36) and EuroQoL-5D (EQ-5D)
- Time to regaining independence in Activities of Daily Living (ADL)
- Resumption of work and other activities of daily living
- Range of Motion of the wrist at both sides
- Radiographic evaluation
- Complications
- Secondary interventions
- Health care consumption with associated costs
- Productivity loss with associated costs

The Patient-Rated Wrist Evaluation (PRWE) measures functional outcome in patients diagnosed with a distal radius fracture [24]. In this 15-item questionnaire, patients are asked to rate the level of pain and disability in their wrist from zero (no pain/difficulty) to 10 (worst pain/unable to do) [25, 26]. The PRWE consists of a 5-item Pain subscale, a 6-item Specific Activities subscale, and a 4-item Usual Activities subscale. Responses to the individual subscales are summed. The total score for wrist pain and disability (100 points) is computed by dividing the sum of the ten functional items by two and adding that subtotal (50 points) to the score for the pain subscale (50 points). This provides a score that ranges from 0 (no pain or disability) to 100 (high level of pain or disability). A validated Dutch version is available [27].

Pain level will be assessed using a 100 mm Visual Analog Scale (VAS), ranging from zero (no pain) to 100 (the worst pain possible). Scores for the affected and contralateral side will both be recorded. At each follow-up visit, patients will also be requested to list the type and quantity of analgesics taken.

The Short Form-36 (SF-36) is a validated generic health survey. The 36 items represent eight health domains, which are combined into a Physical Component Summary (PCS) and a Mental Component Summary (MCS) [21, 22, 28–30]. The eight health domains are physical functioning (PF; 10 items), role limitations due to physical health (RP; 4 items), bodily pain (BP; 2 items), general health perceptions (GH; 5 items), vitality, energy, or fatigue (VT; 4 items), social functioning (SF; 2 items), role limitations due to emotional problems (RE; 3 items), and general mental health (MH; 5 items). The score for each domain ranges from 0 to 100 points. They are normalized to and compared with norm scores of the general population of the United States (1998). Herein, each scale has an average of 50 points and a standard deviation of 10 points. A lower SF-36 score indicates poorer quality of life. A validated Dutch version is available [31].

The EuroQol-5D (EQ-5D) questionnaire is validated for measuring health-related quality of life [21, 22, 32, 33]. EQ-5D is recommended as quality of life instrument for trauma patients [34, 35]. It consists of two parts. The EQ-5D descriptive system has five questions (scored 1–3) regarding problems with mobility, self-care, usual activities, pain/discomfort, and anxiety/depression. The resulting scores are converted to a utility score that ranges from zero to one; a lower score reflects a poorer quality of life. The EQ VAS is a self-rated health status measure, consisting of a vertical 100 mm visual analog scale from zero to 100. A validated Dutch version is available [33].

The time to regaining ADL independence will be asked as a single question. The level of work/ADL resumption will be measured using a numeric rating scale.

The range of motion (ROM) of both wrists (i.e., palmar flexion and dorsal extension, ulnar deviation and radial deviation, pronation and supination) will be measured using a goniometer. The loss of ROM will be calculated as ROM _affected side_ - ROM _contralateral side_.

Radiographic evaluation will be done using routinely obtained radiographs. The degree of radial inclination, volar/dorsal tilt and ulnar variance, the amount of comminution, and the radial length will be measured directly in the digital Picture Archiving and Communication System (PACS) of the participating hospital on standard AP, lateral carporadial, or lateral wrist X-rays. Radiographs will be made using a standardized protocol; 1) for the AP radiographs, the shoulder should be held in 90° abduction, the elbow in 90° flexion, and the wrist in neutral position; 2) for the lateral X-rays, the shoulder should be held in a neutral position and the elbow in 90° flexion; 3) for the lateral carporadial radiographs, the lower arm should be positioned on a 20–25° angled wedge.

Radiographic healing and alignment of the distal radius will be evaluated using X-rays. The treating physician and two independent experts will assess the Lidström score for the repeated X-rays of the wrist blinded from the first assessment [36]. X-rays will be blinded and radiographic evaluation will be done by two experts independently. Any discrepancy will be resolved in a consensus meeting.

The following complications will be collected from the patients’ hospital records; 1) skin problems (threatened skin, necrosis); 2) Surgical site infection [37]; 3) radial nerve pathology; 4) vascular injury (hemorrhage, injury to the radial or ulna artery); 5) tendon pathology (abductor pollicis brevis/longus, extensor carpi radialis brevis/longus, extensor pollicis longus); 6) compartment syndrome; 7) secondary dislocation (loss of length, angulation, or rotation); 8) implant failure (balloon breakage); 9) non-union/pseudarthrosis (i.e., no healing or progress towards healing visible at 6 months after surgery); 10) malalignment severe enough to treat surgically; 11) CRPS type I; 12) systemic, generic or other complications.

Secondary intervention within 1 year of surgery will be collected from the patients’ hospital records. Only interventions aimed at relieving pain, treating infection, promoting fracture healing, or improving function will be recorded. They will be categorized as 1) drug treatment (oral or intraveneous antibiotics, other); 2) surgical treatment (incision and drainage, revision osteosynthesis (plate osteosynthesis, intramedullary nail or other), implant removal, other).

A custom-made questionnaire will be designed to collect details of health care consumption and work absenteeism (i.e., production loss). Health care use associated with diagnosis, treatment, and rehabilitation will be collected. This includes medical specialist care, hospitalization, medication, nursing care, physical therapy, and general practice care [21].

The following data will be collected in order to describe the population and intervention:

- Patient characteristics: age, gender, American Society of Anesthesiologists’ (ASA) classification, tobacco use, comorbidities, medication use, dominant side, sports activities prior to injury, work prior to injury, household composition, and ADL pre-injury.
- Injury characteristics: mechanism of injury, affected side, AO fracture classification [38], and additional injuries.
- Intervention characteristics: time between injury and surgery, length and diameter of PET balloon used, duration of surgery, peroperative complications, admission duration at the Intensive Care Unit and in hospital, type and duration of immobilization (including the use of a sling or collar and cuff), time between trauma and the first physical therapy session, and the duration of physical therapy *(*i.e., total number of sessions).

### Study procedures

Patients will be followed for 1 year. Follow-up visits are scheduled at 2 weeks, 6 weeks, 3 months, 6 months, and 12 months after index surgery. These visits are standard of care for patients with a distal radius fracture. Table 1 shows an overview of the follow-up schedule, including the accepted windows around each visit.

**Table 1:** Schedule of events

Radiographs & Events	Screening	Enrolment	Baseline	Per/Post	2 weeks	6 weeks	3 months	6 months	12 months
				surgery	(10–16 days)	(5–7 weeks)	(11–15 weeks)	(5–7 months)	(12–14 months)
X-Ray^a^	X				(optional)	X	X	X	(optional)
Screening	X								
Informed Consent		X							
Baseline Data			X						
Surgical Report Form				X					
Clinic FU					X	X	X	X	X
Range of Motion					X	X	X	X	X
DASH					X	X	X	X	X
PRWE					X	X	X	X	X
Pain (VAS)					X	X	X	X	X
SF-36					X	X	X	X	X
EQ-5D					X	X	X	X	X
ADL independence					X	X	X	X	X
Work/ADL resumption					X	X	X	X	X
Complications					X	X	X	X	X
Secondary Interventions					X	X	X	X	X
Health Care Use					X	X	X	X	X
Early Withdrawal					^b^	^b^	^b^	^b^	^b^

^a^The AP and lateral X-rays will be used for determining radiographic healing

^b^Only applicable at time of withdrawal

During these regular clinical follow-up visits, AP and lateral radiographs are made for determining radiographic healing and malalignment.

At each planned visit, the clinical investigator (or a research assistant) will document complications and secondary interventions, and will check information within the patients’ hospital files. At the last visit, all planned secondary interventions will also be recorded.

At each planned visit, a physician, clinical investigator, or research assistant will measure the range of motion of the wrist using a goniometer. In addition, patients will be requested to complete a set of questionnaires (DASH, PRWE, VAS for pain, SF-36, EQ-5D, ADL/work, health care consumption, and production loss).

### Sample size calculation

For case series, a generally accepted assumption is that introducing and implementing a (relatively new) surgical technique requires 30 patients [39]. A formal sample size calculation for this study is based on the DASH score (primary outcome measure). The minimal important change for the DASH score is 10 points [19]. Based on literature data we expect a mean DASH of 23 (SD 19) in non-operatively treated patients [40, 41]. In order to detect a 10-point improvement (mean 13, SD 10) in patients treated with IlluminOss® with a two-sided α of 0.05 and a 90 % power (β = 0.10), at least 38 patients are needed. Anticipating 10–15 % loss to FU and 10 % mortality, 50 patients will be enrolled.

### Statistical analysis

The Statistical Package for the Social Sciences (SPSS) version 21 or higher (SPSS, Chicago, Ill., USA) will be used for statistical analysis. The STrengthening the Reporting of OBservational studies in Epidemiology (STROBE) guidelines will apply to data reporting. If necessary, missing values will be replaced using multiple imputation following the predictive mean matching method, using ten imputations. The Shapiro-Wilk test will be used for testing normality of continuous data. All statistical tests will be two-sided, and a p-value <0.05 will be used as threshold of statistical significance.

Descriptive analysis will be performed in order to report baseline and outcome data. Data will be reported as mean with SD (continuous, parametric data), as median with first and third quartile (continuous, non-parametric data), or as number with frequency (categorical data). A Wilcoxon Signed Rank test will be applied for comparing continuous outcome scores at 1 year with those at their first measurement.

Depending on the variability of covariates, multivariable analysis may be performed. A linear mixed-effects regression model will be made to model the relation between specific covariates and the outcome measures that are recorded repeatedly (i.e., DASH and other PROMs). This multilevel model will include fixed effects for covariates like involvement of the dominant side, gender, and age if deemed necessary. These confounders will be selected based upon literature data and by eyeballing the descriptive statistics. The beta values will be reported with their 95 % confidence interval and p-value. Estimated marginal mean will be computed and reported per follow up moment.

For the continuous data determined only once (e.g., time to radiographic healing), a multivariable linear regression model may be developed, with the outcome measure as dependent variable. Potentially confounding variables will be entered as covariate. Beta values will be reported with 95 % confidence interval and p-value.

For the categorical data, a multivariable logistic regression model will be developed, with the outcome measure as dependent variable. Potentially confounding variables will be entered as covariate. Odds ratio’s will be reported with 95 % confidence interval and p-value.

The economic evaluation will include costs for health care as well as for lost productivity. Direct and indirect, medical and non-medical cost will be measured as indicated in the Dutch guidelines for economic evaluations, using standard, published cost prices where possible [42]. Costs for lost productivity will also be calculated.

## Discussion

Despite the frequent occurrence of distal radius fractures, treatment is still at debate. The ultimate goal of treating a distal radius fracture is to restore full wrist function as early as possible. Full range of motion in the wrist declines with disuse and the introduction of early motion following a fracture is critical for the early restoration of normal function, but also for regaining ADL independence. Especially the latter is considered the main strength of the IlluminOss® System. The IO-Wrist study is aimed at determining the effect of operative treatment of distal radius fractures using the IlluminOss® System. Treating patients with this device is expected to result in good and fast functional recovery (i.e., low DASH and high PRWE scores). When comparing outcome with published data on non-operative treatment (i.e., closed reduction and 4–6 weeks plaster cast immobilization, with surgical intervention only if secondary dislocation occurs), we expect similar outcome at 1 year post-injury. However, improved DASH scores are expected to be noticeable until 6 months after treatment initiation, reflecting faster functional recovery in the operative group. Patients operated with IlluminOss® are expected to regain independence sooner and therefore to consume less health care. Improved recovery as well as less total health care use would justify higher costs for the initial surgery. As far as we know, this is the first high-quality study that will evaluate outcome after treating patients with an extra-articular distal radius fracture operated with the IlluminOss® System, using patient-reported, clinical, and economic outcomes.

Four hospitals in the Netherlands participate in this study. The first patient was included on August 31, 2015. Inclusion is expected to be completed within a year. With a follow-up of 1 year data presentation is expected by the end of 2017.

## Abbreviations

ADL: activities of daily living
AO: arbeitsgemeinschaft für osteosynthesefragen (German for “Association for the Study of Internal Fixation”)
AP: anteroposterior
ASA: American Society of Anesthesiologists
BP: bodily pain
DASH: disabilities of the arm, shoulder and hand score
ED: emergency department
EQ-5D: euroQol-5D
GH: general health perception
MCS: mental component summary
MH: general mental health
MREC: Medical Research Ethics Committee
NTR: Netherlands Trial Registry (in Dutch: Nederlands Trial Register)
OTA: Orthopaedic Trauma Association
PACS: picture archiving and communication system
PCS: physical component summary
PET: polyethylene terephthalate
PF: physical functioning
PROMs: patient-reported outcome measures
PRWE: patient-rated wrist evaluation
QoL: quality of life
RE: role limitations due to emotional problems
ROM: range of motion
RP: role limitations due to physical health
SD: standard deviation
SF: social functioning
SF-36: short form-36
SPSS: statistical package for the social sciences
STROBE: strengthening the reporting of observational studies in epidemiology guidelines
VAS: visual analog scale
VT: vitality, energy, or fatigue
WMO: medical research involving human subjects act (in Dutch: Wet Medisch-wetenschappelijk Onderzoek met Mensen)
